# Typhoid Fever in Infancy and Childhood

**Published:** 1903-10-17

**Authors:** 


### TYPHOID FEVER IN INFANCY AND CHILDHOOD.

In a paper on this subject read before the New York Academy of Medicine, Dr. J. P. Crozer Griffith [1] called attention to the fact that, although not by any means so frequent as in later life, yet cases of typhoid fever do occur in infants much more often than has been commonly supposed. With greater care in observation, and especially by the aid of the serum reaction, cases are now diagnosed as typhoid which formerly would have been classed under some other heading. The infection may be conveyed to infants in the milk with which they are fed, as has been shown in a number of epidemics of typhoid which have been traced to a contaminated milk supply, or in water with which their milk is diluted, or which is given to them by itself. A number of cases, too, have been recorded in which the disease was clearly contracted in fœtal life, the bacillus typhosus having been cultivated from the infant's blood shortly after death. As compared with the symptoms of the disease as seen in adult life the two chief characteristics of typhoid fever in infancy and childhood, according to Dr. Griffith's observations are: (1) the less typical onset and course, and (2) the tendency of nervous symptoms to overbalance intestinal ones. It is especially in early childhood that the diagnosis is difficult. The onset is usually slow and insidious, even more so than in adult life, and it may be only after several examinations that the finding of rose spots or enlarged spleen, or the discovery of a Widal reaction leads to a proper recognition of the condition. Where there is doubt a count of the leucocytes may be of material assistance. Occasionally, on the other hand, the onset is quite sudden. Vomiting is a comparatively frequent initial symptom in childhood, although it is rarely very troublesome. Some degree of cough is common, with the presence of coarse mucous râles in the chest. Abdominal pain is not infrequent, but tympanites is not often of sufficient degree to be distressing and to require special treatment. Rose spots are present in the majority of cases and the spleen is probably always enlarged. Although the nervous symptoms are in greater evidence as a rule than the intestinal ones yet this is only the expression of a comparison between the two symptoms. The nervous symptoms themselves in children are not such a striking feature of typhoid as they are in the adult. On the contrary the coma, subsultus, dry brown tongue, muttering delirium, and other symptoms of the advanced typhoid state commonly seen in adults are much less frequently seen in children. A slight delirium, especially at night, is common, but the severer forms of nerve disturbance are usually absent. With regard to the intestinal lesions there is a much less tendency to ulceration than is the case in later life, and when it does occur it tends to be superficial only. The diagnosis of typhoid in infancy is difficult. The tendency for any catarrhal state of the intestine to be accompanied by fever is a disturbing factor. Meningitis is often difficult to exclude in the early stages. Influenza offers peculiar difficulties. There is in it, as in typhoid fever, the continuation of pyrexia without discoverable cause sufficient to explain it. A leucocyte count helps towards the differentiation from many other conditions, but not in distinguishing between enteric and influenza. The serum reaction in such a doubtful case must be relied upon to settle the question. Complications are of less frequent occurrence than in adult life, and hæmorrhage from the bowel and perforation are uncommon. The prognosis of typhoid in children is good as a whole. Thus in 432 cases there were 23 deaths, a mortality rate of 5·32 per cent. But in infants under $2\frac{1}{2}$ years of age this does not hold good, the case mortality being as high as 51 per cent.

[1] Medical News, Sept. 26th.

